# Editorial: Tumor-associated antigens and their autoantibodies, from discovering to clinical utilization

**DOI:** 10.3389/fonc.2022.970623

**Published:** 2022-07-20

**Authors:** Jianying Zhang, Xiangqian Guo, Bilian Jin, Qing Zhu

**Affiliations:** ^1^ Department of Biological Sciences, The University of Texas at El Paso, El Paso, TX, United States; ^2^ Department of Preventive Medicine, Institute of Biomedical Informatics, Bioinformatics Center, Henan Provincial Engineering Center for Tumor Molecular Medicine, School of Basic Medical Sciences, Academy for Advanced Interdisciplinary Studies, Henan University, Kaifeng, China; ^3^ Cancer Center, Dalian Medical University, Dalian, China; ^4^ West China Hospital, Sichuan University, Chengdu, China

**Keywords:** cancer, tumor-associated antigen, autoantibody, biomarker, immunodiagnosis

The detection of autoantibodies in human sera can be used as an important indicator for the diagnosis of autoimmune diseases and has been widely used in clinical research and clinical practice. Researchers in cancer field have found that various types of autoantibodies against cell self-antigens also exist in the sera from cancer patients (1, 2). These antigens related to tumorigenesis are called tumor-associated antigen (TAA). Through the study of the molecular structure and function of some human autoantigens, it is found that there are a wide variety of cellular autoantigens that induce autoantibody responses. Therefore, these cancer-associated autoantibodies might be used as biomarkers for immunodiagnosis of certain type of cancer, or as a tool to monitor therapy as well as as an indicator to predict disease prognosis (3–9). Different approach and technology, including serological analysis of recombination cDNA expression libraries (SEREX) and proteomics, have been extensively used in the identification of TAAs in cancer (10, 11). A bunch of proteins in cancer and pre-cancer conditions were identified and characterized (12–17). More recently, we have noticed that some of other approaches were also used to identify TAAs and detect anti-TAAs autoantibodies, such as whole genome derived peptide arrays and proteome microarray technology (18–20).

In recent two decades, major developments have been made in the field of research on TAAs and anti-TAAs autoantibodies, and many studies have demonstrated that serum anti-TAAs autoantibodies can be used as effective biomarkers for cancer immunodiagnosis (1–9). The diagnostic value, clinical utility, and pathogenic significance of TAAs or anti-TAAs autoantibodies are the focus of ongoing research. This special issue mainly focus on the recent studies associated with the idea and possibility that identification of TAAs and their anti-TAAs autoantibodies can be useful for cancer immunodiagnosis and cancer immunotherapies.

In this Research Topic, our guest editors have invited investigators to contribute original research articles as well as review articles which were mainly related to TAAs and anti-TAAs autoantibodies in cancer immunodiagnosis and cancer immunotherapies, and assembled the current Research Topicfor updating the recent advances in this field. In this special issue, we have received 29 submitted manuscripts, and 18 manuscripts with 139 authors have been accepted for publication. For example, a paper of Jiang et al. used a protein array technology and identify a panel of anti-TAAs autoantibodies in the early detection of lung cancer; a paper of Liu et al. used neoantigen reactive T cells combined with tomotherapy to treat a patient with advanced HCC, who reached a long time progress free survival; a paper of Qiu et al. has evaluated the diagnostic value of autoantibody against PDLIM1 for improving the detection of ovarian cancer; a study from Wang et al. has tested and validated anti-14-3-3 zeta autoantibody might be a biomarker for predicting hepatocarcinogenesis; a study from Lu et al. suggests that the combined application of PD-1-based immunotherapy and anti-cancer drugs has become a new expectation for clinical treatment of colorectal cancer. In addition to these original research papers, several review articles were also included in this Research Topic. For example, a review article from Zhang et al. has summarized the latest advances in the classification of immunotherapy and the process of classification, identification and synthesis of tumor-specific neoantigens, as well as their role in current cancer immunotherapy; a review article from Li et al. has provided an overview of the tumor-associated antigens and anti-TAAs autoantibodies as biomarkers in the immunodiagnosis of steosarcoma; a review article from Jin and Wang has proposed a concept of immunogenic cell death (ICD)-based cancer vaccines and summarized sources of ICD-based cancer vaccines and their challenges, which may broaden the understandings of ICD and cancer vaccines in cancer immunotherapy.

In summary, this Research Topic covers many important aspects in cancer immunology, especially relating to TAAs and anti-TAAs autoantibodies. This Research Topicalso includes recent advances in the basic and clinical studies relating to cancer immunodiagnosis and cancer immunotherapy. We hope that this special issue can provide useful and helpful information to investigators in this field.

## Author contributions

JZ and XG have written the manuscript and BJ and QZ have revised the manuscript. All authors contributed to the article and approved the submitted version.

## Funding

This editorial was supported by the Project of Higher Interdisciplinary Research Institute of Henan University (No. Y21008L).

## Conflict of interest

The authors declare that the research was conducted in the absence of any commercial or financial relationships that could be construed as a potential conflict of interest.

## Publisher’s note

All claims expressed in this article are solely those of the authors and do not necessarily represent those of their affiliated organizations, or those of the publisher, the editors and the reviewers. Any product that may be evaluated in this article, or claim that may be made by its manufacturer, is not guaranteed or endorsed by the publisher.

## References

[1] Tan EM Zhang JY. Autoantibodies to tumor-associated antigens: reporters from the immune system. Immunol Rev (2008) 222:328–40. doi: 10.1111/j.1600-065X.2008.00611.x PMC2719766 18364012

[2] Zhang JY Tan EM. Autoantibodies to tumor-associated antigens as diagnostic biomarkers in hepatocellular carcinoma and other solid tumors. Expert Rev Mol Diagn (2010) 10(3):321–8. doi: 10.1586/erm.10.12 PMC2896686 20370589

[3] Lubin R Zalcman G Bouchet L Tredaniel J Legros Y Cazals D. Serum p53 antibodies as early markers of lung cancer. Nat Med (1995) 1(7):701–2. doi: 10.1038/nm0795-701 7585154

[4] Soussi T. p53 antibodies in the sera of patients with various types of cancer: A review. Cancer Res (2000) 60(7):1777–88. 10766157

[5] Zhang JY Casiano CA Peng XX Koziol JA Chan EKL Tan EM. Enhancement ot antibody detection in cancer using panel of recombinant tumor-associated antigens. Cancer Epidemiol Biomarkers Prev (2003) 12(2):136–43. 12582023

[6] Zhang JY Megliorino R Peng XX Tan EM Chen Y Chan EKL. Antibody detection using tumor-associated antigen mini-array in immunodiagnosing human hepatocellular carcinoma. J Hepatol (2007) 46(1):107–14. doi: 10.1016/j.jhep.2006.08.010 PMC2706782 17067715

[7] Chen Y Zhou Y Qiu S Wang K Liu S Peng X-X. Autoantibodies to tumor-associated antigens combined with abnormal alpha-fetoprotein enhance immunodiagnosis of hepatocellular carcinoma. Cancer Lett (2010) 289(1):32–9. doi: 10.1016/j.canlet.2009.07.016 PMC2823929 19683863

[8] Dai L Ren P Liu M Imai H Tan EM Zhang JY. Using immunomic approach to enhance tumor-associated autoantibody detection in diagnosis of hepatocellular carcinoma. Clin Immunol (2014) 152(1-2):127–39. doi: 10.1016/j.clim.2014.03.007 PMC4096568 24667685

[9] Zhu Q Liu M Dai L Ying X Ye H Zhou Y. Using immunoproteomics to identify tumor-associated antigens (TAAs) as biomarkers in cancer immunodiagnosis. Autoimmun Rev (2013) 12(12):1123–8. doi: 10.1016/j.autrev.2013.06.015 PMC3789855 23806562

[10] Chen Y-T Gure AO Scanlan MJ. Serological analysis of expression cDNA libraries (SEREX) - an immunoscreening technique for identifying immunogenic tumor antigens. In: Su GH, editor. Methods in molecular medicine (2005). Clifton, New Jersey, USA: Humana Press. p. 207–16. 15542909

[11] Le Naour F Brichory F Beretta L Hanash SM. Identification of tumor-associated antigens using proteomics. Technol Cancer Res Treat (2002) 1(4):257–62. doi: 10.1177/153303460200100406 12625784

[12] Imai H Chan EKL Kiyosawa K Fu XD Tan EM. Nuclear autoantigen with splicing factor motifs identified with antibody from hepatocellular carcinoma. J Clin Invest (1993) 92(5):2419–26. doi: 10.1172/JCI116848 PMC288425 8227358

[13] Zhang JY Chan EKL Peng XX Tan EM. A novel cytoplasmic protein with RNA-binding motifs is an autoantigen in human hepatocellular carcinoma. J Exp Med (1999) 189(7):1101–10. doi: 10.1084/jem.189.7.1101 PMC2193003 10190901

[14] Looi KS Nakayasu ES de Diaz RA Tan EM Almeida IC Zhang JY. Using proteomic approach to identify tumor-associated antigens as markers in hepatocellular carcinoma. J Proteome Res (2008) 7(9):4004–12. doi: 10.1021/pr800273h PMC2680441 18672925

[15] Zhang J Wang K Zhang J Liu SS Dai L Zhang JY. Using proteomic approach to identify tumor-associated proteins as biomarkers in human esophageal squamous cell carcinoma. J Proteome Res (2011) 10(6):2863–72. doi: 10.1021/pr200141c PMC3119842 21517111

[16] Peng B Huang X Nakayasu ES Petersen JR Qiu S Almeida IC. Using immunoproteomics to identify alpha-enolase as an autoantigen in liver fibrosis. J Proteome Res (2013) 12(4):1789–96. doi: 10.1021/pr3011342 PMC3743961 23458688

[17] Dai L Li J Xing M Sanchez TW Casiano CA Zhang J-Y. Using serological proteome analysis to identify serum anti-nucleophosmin 1 autoantibody as a potential biomarker in european-american and african-american patients with prostate cancer. Prostate (2016) 76(15):1375–86. doi: 10.1002/pros.23217 27418398

[18] Yang L Wang J Li J Zhang H Guo S Yan M. Identification of serum biomarkers for gastric cancer diagnosis using a human proteome microarray. Mol Cell Proteomics (2016) 15(2):614–23. doi: 10.1074/mcp.M115.051250 PMC4739676 26598640

[19] Pan J Song G Chen D Li Y Liu S Hu S. Identification of serological biomarkers for early diagnosis of lung cancer using a protein array-based approach. Mol Cell Proteomics (2017) 16(12):2069–78. doi: 10.1074/mcp.RA117.000212 PMC5724172 29021294

[20] Yan Y Sun N Wang H Kobayashi M Ladd JJ Long JP. Whole genome-derived tiled peptide arrays detect prediagnostic autoantibody signatures in non-small-cell lung cancer. Cancer Res (2019) 79(7):1549–57. doi: 10.1158/0008-5472.CAN-18-1536 PMC6445725 30723114

